# Effectiveness of a game-based educational strategy e-EDUCAGUIA for implementing antimicrobial clinical practice guidelines in family medicine residents in Spain: a randomized clinical trial by cluster

**DOI:** 10.1186/s12909-022-03843-4

**Published:** 2022-12-24

**Authors:** Isabel del Cura-González, Gloria Ariza-Cardiel, Elena Polentinos-Castro, Juan A. López-Rodríguez, Teresa Sanz-Cuesta, Jaime Barrio-Cortes, Blanca Andreu-Ivorra, Ricardo Rodríguez-Barrientos, José F. Ávila-Tomas, Elisa Gallego-Ruiz-de-Elvira, Cristina Lozano-Hernández, Jesús Martín-Fernández, Educaguia Group, Alberto López García-Franco, Amaya Azcoaga-Lorenzo, Angel Alberquilla Menéndez-Asenjo, Araceli Garrido Barral, Aurora Fernández Moreno, Beatriz Medina Bustillo, Begoña Román Crespo, Elisa Ceresuela Weismann, Emilio Cervera Barba, Jesús Redondo Sánchez, José M. Molero-García, Lizzy Paola Cisneros Almeida, Luis Sánchez Perruca, Luisa María Cabello Ballesteros, Paloma Casado Pérez, Manuel Del Álamo Rodríguez, María Teresa Rodríguez Monje, Mariel Morey Montalvo, Marta Sánchez-Celaya del Pozo, Milagros Rico Blázquez, Luis García Olmos, Raul Ferrer-Peña, Rocío Álvarez Nido, Rosario Riesgo Fuertes, Silvia Pulido Fernández, Sofía Garrido Elustondo, Virginia Hernández-Santiago

**Affiliations:** 1 Research Unit, Primary Care Assistance Management, Madrid Health Service Madrid, C/ San Martín de Porres, 6 _ 5ª Planta, 28035 Madrid, Spain; 2 Department of Medical Specialties and Public Health, School of Health Sciences, Rey Juan Carlos University, Madrid, Spain; 3 Research Network Health Services in Chronic Diseases (REDISSEC) & Research Network RICORS-RICAPP. ISCIII, Madrid, Spain; 4 Instituto de Investigación Sanitaria Gregorio Marañon, IiSGM, Madrid, Spain; 5 Multiprofessional Family and Community Care Teaching Unit Madrid, Madrid, Spain; 6 General Ricardos Health Center, Primary Care Assistance Management, Madrid Health Service, Madrid, Spain; 7 Primary Health Care Research and Innovation Foundation. FIIBAP, Madrid, Spain; 8 Universidad Camilo José Cela, Madrid, Spain; 9 Preventive Medicine Unit, Alcorcon Foundation University Hospital, Alcorcón, Madrid Health Service, Madrid, Spain; 10 Santa Isabel Health Center, Primary Care Assistance Management, Madrid Health Service Leganes, Madrid, Spain; 11 Servicio de Medicina Preventiva, Hospital Infantil Universitario Niño Jesús, Madrid Health Service, Madrid, Spain

**Keywords:** Health Personnel/education, Professional Competence, Experimental Games, Problem Solving, Practice Guidelines, Game-based learning

## Abstract

**Background:**

Clinical practice guidelines (CPGs) have teaching potential for health professionals in training clinical reasoning and decision-making, although their use is limited. The objective was to evaluate the effectiveness of a game-based educational strategy e-EDUCAGUIA using simulated clinical scenarios to implement an antimicrobial therapy GPC compared to the usual dissemination strategies to improve the knowledge and skills on decision-making of family medicine residents. Additionally, adherence to e-EDUCAGUIA strategy was assessed.

**Methods:**

A multicentre pragmatic cluster-randomized clinical trial was conducted involving seven Teaching Units (TUs) of family medicine in Spain. TUs were randomly allocated to implement an antimicrobial therapy guideline with e-EDUCAGUIA strategy ( intervention) or passive dissemination of the guideline (control). The primary outcome was the differences in means between groups in the score test evaluated knowledge and skills on decision-making at 1 month post intervention. Analysis was made by intention-to-treat and per-protocol analysis. Secondary outcomes were the differences in mean change intrasubject (from the baseline to the 1-month) in the test score, and educational game adherence and usability. Factors associated were analysed using general linear models. Standard errors were constructed using robust methods.

**Results:**

Two hundred two family medicine residents participated (104 intervention group vs 98 control group). 100 medicine residents performed the post-test at 1 month (45 intervention group vs 55 control group), The between-group difference for the mean test score at 1 month was 11 ( 8.67 to 13.32) and between change intrasubject was 11,9 ( 95% CI 5,9 to 17,9). The effect sizes were 0.88 and 0.75 respectively. In multivariate analysis, for each additional evidence-based medicine training hour there was an increase of 0.28 points (95% CI 0.15–0.42) in primary outcome and in the change intrasubject each year of increase in age was associated with an improvement of 0.37 points and being a woman was associated with a 6.10-point reduction. 48 of the 104 subjects in the intervention group (46.2%, 95% CI: 36.5–55.8%) used the games during the month of the study. Only a greater number of evidence-based medicine training hours was associated with greater adherence to the educational game ( OR 1.11; CI 95% 1.02–1.21).

**Conclusions:**

The game-based educational strategy e-EDUCAGUIA shows positive effects on the knowledge and skills on decision making about antimicrobial therapy for clinical decision-making in family medicin residents in the short term, but the dropout was high and results should be interpreted with caution. Adherence to educational games in the absence of specific incentives is moderate.

**Trial registration:**

ClinicalTrials.gov Identifier: NCT02210442. Registered 6 August 2014.

**Supplementary Information:**

The online version contains supplementary material available at 10.1186/s12909-022-03843-4.

Contributions to the literature

- A game-based educational strategy appears to be more effective in the short term than classic dissemination strategies for the implementation of clinical practice guidelines in the postgraduate training program of family and community medicine
- Previous training in evidence-based medicine is related to higher skill acquisition after clinical practice guideline implementation processes in the residency setting
- The effectiveness of the intervention has been tested with an appropriately designed pragmatic clinical trial.
- Adherence to educational games in the absence of specific incentives is moderate.

## Background

Several studies have analysed the potential teaching of Clinical practice guidelines (CPGs) for training clinical reasoning and decision-making for medical residents in different clinical areas, such as hypertension [1], lower urinary tract infection treatment [2], approach to risk factors for chronic kidney disease [3], colon cancer screening [4] and the selection of an oral anticoagulant to prevent ischaemic accidents [5]. However, their implementation continue to be deficient [6–8].

The implementation of a CPG aims to ensure that the recommendations it proposes are followed [9]. The most used strategy for promoting awareness of CPGs in training programs for medical residents is passive dissemination [10], sometimes accompanied by reminders [11] or incentives. However, these methods have obtained only moderate effects [12]. Other simple strategies, such as the organization of seminars in which the contents of the CPG are presented, offer little evidence of effectiveness when the outcomes of the care process are measured [13]. Reading clubs can have some, improving their knowledge of clinical epidemiology and biostatistics, habits, reading skills and the use of medical literature, including CPGs [10]. More complex interventions that mix training methodologies with quality improvement plans have shown an impact on intermediate outcomes in diabetic patients in a Family medicine residency programme [1].

Some strategies incorporated in recent decades has been educational games and gamification. Both of them are active methodologies to turn the student into the protagonist of his learning, using game dynamics. Game Based Learning can use games, either created or invented for the occasion, in order to learn through them. Thus, the game becomes a vehicle to consolidate concepts [6, 14]. Gamification is a teaching strategy that incorporates elements of game design and its mechanics. It involves the design of a real or virtual educational environment that involves the definition of tasks and activities using the principles of games [15].

Games allow the practical application or simulation of real-life conditions that reflect complexity of clinical practice better than traditional teaching formats and have the potential to motivate students [16, 17]. They have been primarily used with medical students finding positive effects on increasing knowledge and many of the studies were of low or moderate quality and did not evaluate the impact of games on student satisfaction, skills, attitudes or behavior [18–20]. Educational games disseminated by information and communication technologies (ICTs) are well-accepted and are used more if they are fun and if they help develop patient interaction skills.

In medical residents training, game-based learning methodologies are often presented through ICT and can be classified according to two criteria: a) the mechanism of the game—pay games, thinking games, quizzes, role-playing games and simulations/skill games-and b) the complexity of learning experience classified in four levels. The simplest learning process in a game is the experience, in a two-stage game, experience is followed by reflection. A three-stage game model includes experience, reflection, and the creation of a new plan for the next cycle (e.g., in games that allow the player to choose response categories or that allow bidding). A four-stage model includes experience, reflection, abstraction of the topic, and planning the next step of the experience [16].

Some studies show in surgical specialties, educational games are valued as acceptable and useful by physicians [21] and have also been used as an evaluation method [22] but the results on their effectiveness in improving specific skills have contradictory results. [23, 24]. Among family medicine and internal medicine residents, the majority of the studies used the game as a teaching and review tool, and 4% as an assessment tool [25]. Most of them had one or two learning phases [26] and evaluated as main outcomes the improvement of knowledge and skills [27] but evidence of their effectiveness in improving the implementation of CPG is scarce. Most of the ICT-mediated games refer to web sites or software designed for this purpose [28] and in those in which their usability and acceptability among medical residents of different medical specialties have been studied, the evaluation has been good [29].

Different systematic reviews [30–32] conclude that their results do not confirm or refute the usefulness of games as a teaching strategy for health professionals and that there is a need for additional high-quality research to explore the impact of games on patient outcomes and professional performance [33]. The use of educational games in training programmes is conditioned by both logistical factors—as high numbers of students, the need for teacher preparation time, the complexity of developing simulations—which are recognized as crucial for developing the competence of health professionals [34], and individual factors, such as reflective practices and the significant gender dissonance regarding preferred types of games, the educational value of video games and the desire to participate in games that realistically simulate the experience of clinical practice [18].

In Spain, the specialty of Family and Community Medicine, like other specialties, is obtained through a postgraduate training program known as Medical Internship (MIR) with a duration of 4 years and taught in accredited health centers and hospitals. We have an official national training program that includes among the competences the medical resident has to learn during his training period, the acquisition of knowledge about evidence-based medicine tools and clinical practice guidelines. GuíaSalud is the Spanish National Health System’s CPG elaborating institution, created in 2002 as an instrument to improve the quality of healthcare. Its catalog can be consulted at https://portal.guiasalud.es/gpc/. In our study, the antimicrobial therapy guide [34] was prioritized, for its clinical relevance, as the knowledge area in which to study the effectiveness of and adherence to the e-EDUCAGUIA game strategy.

The objective was to evaluate the effectiveness of a game-based educational strategy e-EDUCAGUIA using simulated clinical scenarios to implement an antimicrobial therapy GPC compared to the usual dissemination strategies to improve the knowledge and skills on decision-making of family medicine residents. Additionally, adherence to e-EDUCAGUIA teaching strategy was assessed.

## Methods

### Design and population

A multicentre randomized clinical trial by clusters was conducted within the framework of the EDUCAGUIA Study [35] (Supplementary material 1). This article has been elaborated following the Consort cluster guideline (Supplementary material 2).

Invitations to participate were extended to the 7 multiprofessional family and community care teaching units (TUs) accredited by the Ministry of Health in Madrid region, Spain. In 2015, 844 family medicine residents and 96 family nurse residents were trained in these units, in 131 health centres and 20 associated hospitals [36]. Family medicine residents who had at least 6 months of training were eligible to participate.

### Sample size

Considering that to evaluate the effectiveness of this intervention, 200 volunteer medicine residents would be needed, and based on assumptions about design effects and estimated losses detailed in the study protocol for the general project (see Supplementary material 1) [35], a sample of this size would allow us to detect a somewhat large effect size, on the order of 0.725, with a power of 90%. With a common standard deviation of 20 points for both groups, this sample size would allow us to find mean differences of 14.5 points.

### Recruitment

Recruitment was carried out in two stages. First, those responsible for the 7 TUs in the Community of Madrid were invited to participate voluntarily through a presentation session in which the educational intervention, objectives, and evaluation methodology of the study were described. Subsequently, each UT was responsible for recruiting its medical residents on a voluntary basis. This was done through the ordinary communication channels of the mandatory training plan (e-mail), including a letter of invitation from the principal investigator of the project.

Their participation in the trial fell within the framework of the planned activities of the residency program. In these cases, individuals cannot act independently, and the principle of autonomy is absent [37]. For this reason, and following international examples, the heads of studies, in their role as gatekeepers who act as a “mechanism of representation of the conglomerate”, were asked to consent to participate. The heads of each TU agreed to participate in the study after receiving an acceptance form that provided the details of the educational intervention and how it would be evaluated through the acceptance form. However, each individual had to tacitly consent to participate in the study once the TUs had agreed to participate, as recommended [38].

### Randomization

Randomization was performed after recruitment of medicine residents. The 7 TU were assigned by randomization to the intervention group or the control group by an in- dependent statistician who was masked to the TU identifiers using Epidat 4.1 software. Random allocation was stratified by TU size and done in blocks of two, with intervention and usual care allocated simultaneously. The unit of analysis was the medicine resident.

### Intervention

The educational strategy e-EDUCAGUIA is a complex intervention based on an educational game with a ranking and a combination of competitive play styles. It was designed by a multidisciplinary team of primary care professionals with experience in training students and medical residents and know how to guideline development and use new technologies as teaching tools. Its development followed the recommendations and taxonomy proposed by the Cochrane Effective Practice and Organization of Care Review Group. Supplement 1 [35] details this intervention according to the methodological proposal of Perera et al. [39] and TDIER ( Supplementary material 3).

The game can be described as a contest-type game with two stages (experience followed by reflection) [16]. The contents of the game were developed according to clinical areas and recommendations selected from CPG based on population prevalence criteria and frequent reasons for consultation in primary care. These selections were made by the consensus of a multidisciplinary team. The included content areas were adult infections, lower upper and lower respiratory, upper and lower urinary tract, genital and breast, adult skin and soft tissue, oral and dental, ophthalmological and dental infections.

The material was developed by experts based on the recommendations of the selected guide and the competencies for these areas outlined in the family medicine programme using accredited training material recommended by the Infectious Disease Group of the Madrid Society of Family and Community Medicine For each processes, clinical scenarios were constructed that covered the following dimensions: fundamentals, clinical orientation, diagnosis and therapy. The scenarios and questions were developed. Due to its importance, the therapeutic dimension was prioritized; 50% of the play opportunities focused on this dimension.

Each clinical scenario was developed and discussed by the research team and could include text, images or videos. The questions were introduced after the presentation of each clinical scenario. Some questions about each scenario asked about knowledge and others presented consecutive steps in decision making; for example, in a first question the medical resident had to choose an antibiotic treatment for a specific process and patient profile, in a second question he/she had to provide information about the clinical evolution or preferences of the patient and had to make decisions about requesting additional tests, referral to hospital, change of treatment, etc.). Supplementary material 4 shows as an example some screen games in original version.

A pilot study was conducted with 10 professionals (family physicians and family medicine residents) on 4 different occasions to review scenarios and questions, with their contributions, the questions and rules were modified, and the final version was determined.

The mechanics of the game consist of answering a battery of 10 questions with 4 response options in each area ( a total of 100 questions), and its objective is to accumulate the most points. The maximum time allowed to resolve an area is 6 min. Each correct answer is awarded 10 points, with 3 points awarded for each minute left over at the end of the game and 3 points awarded for each help item that is not used. The 3 help items available are “ guardian help”, which consists of an audio file that reviews the main concepts that the questions covers; the “50%” wild card, which eliminates 2 of the possible answers; and the “guide access wild card”, which contains a direct link to the chapter of the CPG that pertains to the relevant pathology. The difficulty was increased to allow the player to learn the mechanics of the game and obtain benefits.

The application generates a report for each completed training session and ranks the medical residents to allow them to compare their results with those of other users in terms of both the number of successes and in the time required to complete the scenarios. The website is accessible from any computer after registration, and an app allows the game to be downloaded on any smartphone.

In both control and intervention groups, an initial 60-min session was held on what CPGs are and where to find them. The National Guidelines Plan of the Spanish National Health System GUIASALUD, its web page and its CPG library catalogue, including the antimicrobial therapy guideline [40], were presented. After this initial session, a test (pre- test) was administered to assess knowledge and skills on decision making based on 10 clinical scenarios on antimicrobial therapy. After this session both groups received the routine dissemination by e-mail of the CPG and the Intervention group also received an invitation to use educational games designed for this project for a month. During the first week of use of the e-Educaguia application, the participants were consulted on possible improvements, and a modification was included, stopwatch did stop when the participant requested a tutor’s help.

Another face-to-face session was held for both groups one a month after the intervention, and a final test ( post test) was administered. Both sessions were made in the same place, a classroom in each TUs and during their usual meeting timetable from 2 to 3 pm.

### Variables

Primary outcome was the test score evaluated knowledge and skill on decision-making based on 10 clinical scenarios related to the CPG one month after the intervention. It included 25 questions formulated in such a way that they required the integration of knowledge as it is applied to decision-making in clinical practice according to level 2 of the learning proposal of Kirkpatrick et al. [41]. This level represents the extent to which participants change attitudes, improve knowledge and/or increase skills as a result of participating in the programme.

As in the scenarios of the games, half of the questions on the questionnaire were related to therapeutic management. The ranged test scores from 0 to 100 points. The time available to complete it was 45 min and the medical resident could check the scores at the end.

Secondary outcomes were the change of intrasubject in the test score, from basal to month post intervention, the adherence to the EDUCAGUIA strategy ( measured as the percentage of students who had obtained some score on the game and was included in the ranking) and the usability collected with an open question.

The sociodemographic variables age, gender and nationality were collected. Regarding specialized health care training variables, the ranking on the access test (number of order), year of residence and results on the annual residency program evaluation (acceptable, outstanding or excellent) were collected. As measures of previous training related to the number of hours of critical appraisal and training in CPG and evidence-based medicine tools per year of residency.

From the TUs, the following data were collected: total number of medical residents by year of residence; position of the TU among the Regarding specialized health care training rankings in family medicine resident in the last three classes; availability of a training programme for medical residents on evidence-based practice and CPG use in the previous year and the number of hours of the programme; characteristics of the TU’s head of studies and technician, such as gender, age, specialty, academic level (graduate or physician), time in the position, experience in preparing CPGs and experience with EBM tools.

### Data collection

The TUs variable data were provided by the heads of studies and technicians of each unit. The medical residents were given an identification key and access to the project website, where, after agreeing to participate in the study, they provided their sociodemographic and training variables.

The test scores obtained basal and one month post intervention were recorded. Data on each medical resident’s participation in the game, the time and success in its execution were collected from reports prepared by the programme after the completion of the game.

The participants in the intervention group could provide input on the usability of the game in the project website where the game was hosted and by email.

### Statistical analysis

The description of the baseline characteristics, continuous variables are expressed as means and standard deviations (SD), and categorical variables are expressed as proportions with 95% confidence intervals. The tests used to compare variables between groups were the chi-square test or Fisher’s exact test for categorical variables and the student’s t-test or Mann–Whitney test for continuous variables.

Primary outcome analyses were carried out in accordance with the intention-to-treat principle, and per-protocol analysis missing data were replaced with the mean of each TUs at 1 month. The difference in means between groups in the test score after one month of the intervention was calculated using Student’s t-test with its corresponding 95% CI.

To adjust the effect of the intervention and to explain the factors associated to the final test score general linear models—GLM were used as regression methods, in this case with "identity" link functions and the normal or gamma distribution. GLMs allow unbiased estimators of the associations to be obtained in the presence of heteroscedasticity [42]. Standard errors were constructed using robust methods, taking into account the subjects’ origin from different clusters (TUs). Family medicine residents as TUs covariableswere included in these multivariate regression models. We tested several regression models, from which we selected the best ones for their fit criteria (AIC and BIC).

As a secondary outcome the difference intrasubject was calculated using Paired Student t Test and a general linear model was constructed to explain the factors associated with the same methodology as for the primary outcome.

Adherence was calculated as percentage and the factors associated with the adherence game were analyzed using a general linear model with a logit identity function. The usability was collected with an open question and the responses are described.

## Results

### Characteristics of the study participants

**Fig. 1:**
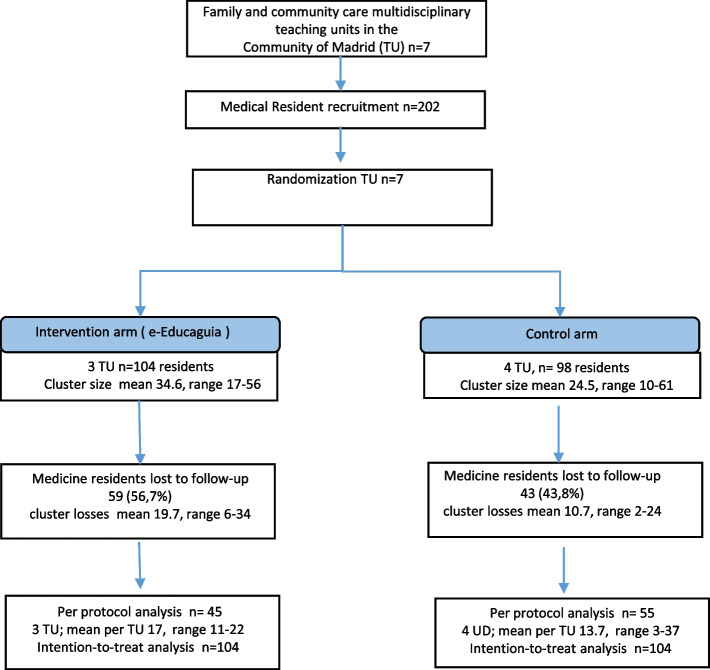
Flow Diagram

A total of 202 medical residents were included in the study. Figure 1 presents the flowchart of the study.

The subjects were equally distributed in terms of gender, age and nationality. There was a nonsignificant tendency for there to be less training in EBM but more training in critical appraisal in the intervention group. The percentages of medical residents in their first two years and those in their second two years were equivalent in both groups. The scores of the medical residents in their first year were better in the intervention group than in the control group. There were no differences in the pre-test scores between the intervention and control groups. Table 1 presents the characteristics of the subjects in each group and their comparability.

**Table 1:** Characteristics of the included subjects and comparison between groups

	**Total** ***n* = 202**	**Intervention group** ***n* = 104**	**Control group** ***n* = 98**	***p***
	% or mean(SD)^a^	% or mean^a^ 95% CI	% or mean^a^ 95% CI	
**Women**	74.75	79.0	69.8	0.168
**Age**^a^	30.70 (5.73)	30.23 (5.34 )	31.10 (6.12)	0.163
**Year of residency**				
R1	24.75	23.1	27.1	0.153
R2	32.67	34.6	31.3	
R3	27.23	31.7	20.1	
R4	15.35	10.6	20.8	
**Spanish nationality**	89.60	92.3	86.4	0.425
**Other speciality**	4.95	4.8	5.1	0.923
**Hours EBM learning**^a^	7.19 (7.56)	6.75 (6.1)	7.97 (8.80)	0.092
**Hours critical reading**^a^	9.63 (10.60)	10.62 (13.24)	8.58 (6.69)	0.087
**CPG sessions**^a^	2.60 (3.29)	2.31 (2.88)	2.90 (3.67)	0.101
**R1 rating**				
Adequate	69.80	64.40	75.0	0.015
Outstanding	18.31	17.3	19.8	
Excellent	11.88	18.20	5.2	
**Basal knowledge test score**^a^	53.61 (9.58)	53.10 (8.99)	54.12 (10.15)	0.227

^a^Mean; *SD* standard deviation, *EBM* Evidence-Based Medicine, *CPG* Clinical Practice Guidelines, *R* Medicine resident

### Primary outcome

At the month post intervention, the post-test was completed by 100 medicine residents, 45 in the intervention group (43.3%) and 55 in the control group (56.1%). No statistical differences were found between included participants and dropouts. Participants per teaching unit are presented in the Supplementary 5.

Difference in means of test score at 1 month was 11 points ( CI 95% 8,67 to 13,32) in ITT analysis and 11.3 points ( CI 95% 6,8 to 15,9) in per protocol analysis. Given that the standard deviation of the distribution of the post-test scores was 12.72 points, the effect sizes can be estimated as 0.88 (Table 2).

**Table 2:** Primary and secondary outcome at one month post intervention

	Intervention group Mean (SD) n()	Control group Mean (SD) n ()	Unadjusted Difference in means 95% CI
**Primary outcome**			
Score at 1 month Per protocol analysis *n* = 100	53.9 (13.8) *n* = 45	42.5 (9,1) *n* = 55	11.3 6.8 to 15.9
Score at 1 month Intention-to-treat analysis *n* = 104	53.7 (9.73) *n* = 104	42.70 (6.89) *n* = 98	11,0 8,67 to 13,32
**Secondary outcome**			
Change from baseline score (T1-T0) Per protocol analysis	0.5 (17.3)	-11.5 (12.4)	11.9 5.9 to 17.95

Belonging to the intervention group was associated with an 11.81-point increase in the mean score adjusted by covariables compared to the control group. Factors associated were the EBM training hours, for each additional hour of EBM training, the mean score increased by 0.28 points. No differences were found in any other variables (Table 3).

**Table 3:** Factors associated with post-test scores at one month after the intervention

	**Coef**	**95% CI**	***p* > z**
**Intervention vs. control**	11.81	5.10 to 18.52	0.001
**Women vs. Men**	-0.80	-3.47 to 1.87	0.559
**Age in years**	0.01	-0.34- to 0.37	0.938
**Year of residency**			
R2 vs R1	-0.94	5.83 to 3.95	0.706 -
R3 vs R1	-0.82	-9.27 to 7.63	0.848
R4 vs R1	0.711	-4.77 to 6.19	0.799
**R1 rating**			
Adequate vs Outstanding	4.24	-0.68 to 9.18	0.091
Excellent vs Outstanding	2.37	-5.48 to 10.22	0.554
**Previous another specialty**	7.88	-2.64 to 18.39	0.142
**EBM tools training hours**	0.28	0.15 to 0.42	< 0.001
**Critical Appraisal training hours**	-0.07	-0.33 to 0.18	0.580
**Use Clinical Practice Guidelines training hours**	-0.28	-1.28 to 0.72	0.581

*N* = 100; link: g(u) = u; F-distribution family (normal); AIC = 7.720

*EBM* Evidence-Based Medicine, *R* Medical resident

### Secondary outcome

Difference in means in the change intrasubject from baseline test score to one month test score was 11.9 (IC 95% 5,9–17,9) (Table 2), being the effect sizes estimated 0.75.

In the multivariable analysis, belonging to the intervention group was associated with an increase of 12.04 points, each year of increase in age was associated with an improvement of 0.37 points and being a woman was associated with a 6.10-point reduction. Each additional hour the mean score increased by 0,13 points but at the limits of statistical significance (Table 4).

**Table 4:** Factors associated with the change intrasubject at one month after intervention

	**Coef**	**95% CI**	***p* > z**
**Intervention vs. control**	12.04	6.80 to 17.28	0.001
**Women vs. men**	-6.10	-7.76 to -4.44	0.001
**Age in years**	0.37	0.01 to 0.73	0.044
**Year of residency**			
R2 vs R1	2.81	-0.26 to 5.90	0.073
R3 vs R1	-0.06	-11.15 to 11.01	0.991
R4 vs R1	0.59	-5.91 to 7.09	0.859
**R1 rating**			
Adequate vs Outstanding	5.25	-2.11 to 12.62	0.162
Excellent vs Outstanding	3.76	-6.21 to 13.73	0.459
**Previous another specialty**	3.25	-17.95 to 24.46	0.764
**EBM tools training hours**	0.13	-0.02 to 0.28	0.081
**Critical Appraisal training hours**	-0.15	-0.37 to 0.08	0.207
**Use CPG training hours**	-0.20	-1.02 to 0.06	0.624

*N* = 97; link: g(u) = u; F-distribution family (normal); AIC = 8.206

*EBM* Evidence-Based Medicine, *CPG* Clinical Practice Guidelines, *R* Medicine resident

In terms of adherence, 48 of the 104 subjects in the intervention group (46.2%, 95% CI: 36.5–55.8%) used the games during the month of the study. Only a greater number of EBM training hours was associated with greater adherence to the educational strategy (OR 1.11; CI 95% (1.02–1.21) Although the hours of critical appraisal were significantly associated with the probability of not having played even though the magnitude of the association was practically negligible (OR 0.98; CI 95% CI 0.97–0.99). Table 5 shows the factors associated with greater adherence.

**Table 5:** Factors associated with the adherence to educational games in the intervention group

	**Odds ratio**	**95% CI**	***p* > z**
**Women vs. men**	1.19	0.66–2.16	0.557
**Age in years**	0.98	0.90–1.07	0.629
**Year of residency**			
R2 vs R1	0.73	0.27–1.98	0.538
R3 vs R1	1.22	0.46–3.19	0.688
R4 vs R1	0.47	0.02–12.43	0.651
**R1 rating**			
Adequate vs Outstanding	1.26	0.60–2.66	0.539
Excellent vs Outstanding	1.49	0.44–5.04	0.518
**Previous another specialty**	1.43	0.31–6.55	0.645
**EBM tools training hours**	1.11	1.02–1.21	0.017
**Critical Appraisal training hours**	0.98	0.97–0.99	0.000
**Use CPG training hours**	0.84	0.57–1.23	0.372

*N* = 104; Link: g(u) = logit (u); F-Distribution family (Binomial); AIC = 1.323

*EBM* Evidence-Based Medicine, *CPG* Clinical Practice Guidelines, *R* Medicine resident

The usability of the tool was studied in the intervention group. The contributions could be classified along three axes: a) Dynamics of the game: the rankings and audio aids were valued very positively. In contrast, the failure of the timer to stop when the player sought help from the tutor was considered a “negative handicap”; this was resolved during the first week of the application’s use. b) Content of the game: the participants considered short, trivia-like formulations that allowed greater dynamism in the game to be attractive and motivating. c) Operation of the application: During the first week, technical problems that hindered access to the game were present.

## Discussion

### Main findings

This paper presents the results of a game-based educational strategy that shows positive effects on the knowledge and skills on decision-making of family medical residents in the short term. Even considering the limitations of this clinical trial, mainly referring to loss to follow-up, we can state that the intervention leads to a situation of clinical management capacity, measured through clinical scenarios, better than that assessed in the control group.

The importance of these results can be determined from the pragmatic nature of the intervention, the reasonable internal validity and the applicability to a large population of medical residents, as evident from the inclusion and exclusion criteria of the study, in the context of very scarce evidence on the effectiveness of this type of strategy. The results are of a non negligible magnitude, with large effect sizes according to Cohen’s classification (0.75–0.88) [43] and a magnitude only slightly lower than those obtained by other studies that explored educational game use in medical students but had designs that were much more subject to bias, such as before-after studies [20].

### Comparison with other studies

Studies that introduced competitive games into medicine resident training in general have measured the improvement of knowledge, finding limited or discrepant effects over time for different subjects [44], or have focused on intermediate results, such as time dedicated to reading [45]. However, the greatest problem of these studies is not the inconsistency of their results but the inappropriateness of their designs for demonstrating the effect of the interventions, as the review by Akl et al. [32] showed.

In that review, the only study with an adequate design for evaluating effectiveness was the one by Burke et al. [33]. That study evaluated the effectiveness of an educational game for health professionals that was based on a television game show (“Family Feud”) and was used as a reinforcement technique to try to improve knowledge in the field of infectious diseases. The study did not evaluate any patient or care process results, as was also the case in our study. The study design was factorial, and of the two interventions tested, the use of the game as a reinforcement strategy improved knowledge after a videotape intervention but not after an intervention consisting of a self-study module. The reason for this differential effect is not clear. The group assigned to the game condition performed better on the knowledge test, although the meaning of this improvement (an average of one point over a range of 20) is not very clear. In our case, the magnitude of the effect allows us to discuss the clinical relevance of the result.

Regarding the factors in addition to the intervention that were related to an improvement in the outcome, the number of hours of EBM training was related to better results. The inclusion of EBM training in a curriculum can improve knowledge not only in areas related to research [46] but in other areas overarching specialty-specific performance in certain groups of medical residents [47]. In Spain, the MFyC training plan places special emphasis on training in these tools [48].

Regarding intrasubject change, all of the subjects had worse results at the end of the test than at the beginning. There are several factors that can explain this finding, since the tests were the same at both time points, although the order of the questions differed. First, in the initial session, the students had more time to take the test for organizational reasons, which may have allowed them to consult sources for answers, a factor that was formally addressed in the post-test evaluation. The change in question order and less time may not be the only reasons for the worsening scores over time. Perhaps the Hawthorne effect (the improvement in scores when feeling observed) had more weight when the first test was announced than later. [49, 50]. Other reason is whether the results cannot be explained simply by differences in forgetting the information learned in the first session. This session presented participants with relevant information about the guideline immediately before the first test was administered. After one month, when the final test was administered, this information would have been forgotten in different degrees depending on whether it has been retrieved/applied during the interval between the two tests. Whereas the game group retrieved it during the game, the retrieval, if any, would be less frequent in the control group (some participants may even have not accessed the material).

Although this finding could imply a criticism of the results compared with studies with other designs, this is not the case; because this phenomenon occurred in a nondifferential manner in both the intervention and control groups, it cannot be assumed to contribute to the magnitude of the difference that was found. The results are consistent with those of previous studies when the differences in the means of the intrasubject changes are evaluated, since they are also explained by the hours of previous EBM training (although in this case, the significance was marginal, *p* = 0.081) and by age and gender. For each year of increase in age, the differences between the final and initial results increased by an average of 0.36 points. Although there is evidence that knowledge/skills decay across professional life, this decrease seems to be especially significant starting at 15 years of professional practice but not in the context of specialization[51, 52], moreover would not apply in the context of a one-month study neither, in this case during the post-graduate training period, the oldest age is usually that of medicine residents in their final years of specialisation.

Women presented a negative intrasubject change of a greater magnitude. This difference found between men and women should be further investigated but it cannot be attributed to the game itself (since the change intrasubject is already adjusted by receiving the intervention). Still we would like to reflect on the importance of taking into account gender and use of games related to type of game, time playing or previous experience, when designing learning strategies based on gamification. There is debate regarding the role of gender in the use of games, and it is considered that men tend to play games more often, and that men and women have significantly different attitudes towards video games favour males [53, 54]. However, some studies conclude that games can be equally effective and motivating for both men and women, suggesting that the impact of gender on acceptance tends to disappear during the implementation phase [55]; furthermore, men and women agreed that they would be more likely to use multiplayer.

### Strengths and limitations

As in all pragmatic designs, we sought to achieve a balance between the generalizability of the results (external validity) and their reliability or accuracy (internal validity) [56]. We tried to minimize systematic bias, which compromises internal validity, through the random assignment of groups, blinding of the random assignment of the teachers of the first session and systematized collection of the results.

One of the greatest limitations is related to dropout near to the 50% To analyze its possible impact we performed complementary analyses. Baseline characteristics were compared between control and intervention group and we didn’t found differences and we analyzed the potential effect of the differential distribution of the confounding factors (when participants who are lost differ in some way from those who are not, which did not seem to be the case in the present study) and show an intervention effect of a magnitude very similar to that of the differences in crude means and the intention-to-treat and per-protocol analysis. The differences in intra-subject change also offered consistent results. These circumstances, without providing any absolute guarantee by themselves, allow us to be confident in the quality of the evidence obtained, which is also assured by the use of a solid design as a pragmatic clinical trial, but it should certainly make us very cautious in our conclusions.

The study was conducted according to the standards of pragmatic clinical trials, so it was not possible to check of the extent to which participants in the control group had actually studied the guide, or if they read only the guide in the beginning but were no longer exposed to the information whereas the intervention group was by engaging with the game throughout the month. This could possibility that it is not the approach (game vs reading material) per se but simply the amount of exposure to the to-be-learned material that accounts for the difference.

The measurement tool used to evaluate competence in our study, although it referred to “pure” knowledge, was formulated in such a way that it required the integration of this knowledge in the way in which it is applied to decision-making in clinical practice. According to the sequence of levels to evaluate the training programmes proposed by Kirkpatrick et al., who propose four levels [41], our tool evaluated knowledge, but its formulation required that these be integrated in a way that allowed immediate decision making. In addition, the evaluation took place in a context of simulated clinical practice, so that, even at level 2, it tried to approach level 3 by addressing the capacity to transfer this knowledge to decision-making in clinical practice (in addition to being measured outside the context of educational activity).

Regarding the generalizability of the results, it cannot be assumed that the family medicine residents who were included in the study are representative of all family medicine residents in the Community of Madrid, but it can be said that the included sample has an age and gender distribution similar to that of all medicine residents in this specialty in Madrid and that the distribution among all years of residence was representative. In addition, the sample included subjects with different qualifications, and the number of training hours was consistent with the complementary training programmes for medicine residents in this specialty [48], which reinforces essential skills that are of special interest in training and research [57].

It is worth asking whether the ability to recruit medicine residents, which is striking in some TUs, can be related to some specific characteristics. It is necessary to point out that some TU directors were involved in the research project, which may have been associated with an increased recruitment capacity. However, to ensure that this situation did not bias the results, we decided to randomize pairs of TUs according to their number of participating medical residents; thus, the proportion of participants from each TU was corrected with randomization. Losses were not proportionally equal for all TUs at follow-up, but these differences were on the verge of statistical significance. That is, circumstances suggested that there was greater recruitment than would be expected from voluntary participation, although this does not represent a limitation in internal validity.

Another factor to consider is the reproducibility of the evaluated strategy. A pragmatic design is associated with a greater capacity for extrapolation in general because it tests strategies instead of simple interventions [58]. The strategy is simple once the game is designed. Although adherence was quite limited in the present study, this may be because there was no incentive for use other than intrinsic motivation. If this strategy were to be incorporated into the usual teaching practice in the TUs, it would be accompanied by additional incentives; in fact, incorporation into the training programme in and of itself would be an incentive because in that case, the use of the strategy would be mandatory and essential to receiving a positive evaluation. In fact, when experiments with educational games have been carried out in academic environments (in which the student is evaluated both as a study participant and academically), adherence rates are substantially higher [20]. However, the greatest incentive for adhering to a game strategy is motivation to play the game, sometimes accompanied by competitiveness [29]. A motivating element of other experiences that was not incorporated into this strategy, is the possibility of competing as part of a group [26, 29].

The usability problems that were outlined can be easily addressed in later versions of the game. This type of game is highly accessible; it can be accessed with a smartphone, which addresses the potential lack of accessibility that has been reported in other studies [29]. At present, technology has a key role in training processes, and this role has been expanded in the context of the pandemic. The traditional strategies that have been used in the attempt to improve medicine residents’ use of CPGs on medical residents have had limited effects.

Studies such as the one presented here present new avenues of educational innovation that deserve to at least be explored and evaluated beyond the proposed experimental framework. There are two initial factors that, considering the results, should be addressed in future research. One is usability; a regulated evaluation must be carried out since the challenge of educational game design is to develop solutions that attract players and, in turn, are educationally effective [59]. For future studies, it would be advisable to include other tools to evaluate usability. At the time when we developed the game and carried out the study, the methodological proposals for its evaluation were more limited than those currently being developed.

Another relevant issue is the durability of the effect over time. The study presents the results of an evaluation at one month, a period that some authors have recommended for assessing results; however, it is known that gains in knowledge and skill acquisition present a constant decline over time, and the objective of training is the long-term maintenance of benefits. In addition, there are differential losses in knowledge over time between traditional training strategies and those based on educational games (in favour of the former) [19], which makes the study of this issue even more relevant.

## Conclusions

The game-based educational strategy e-EDUCAGUIA showed positive effects on the knowledge about antimicrobial therapy for clinical decision-making in family medical residents in the short term, but the dropout was high and results should be interpreted with caution. Adherence to educational games in the absence of specific incentives was moderate.

Having received more hours of EBM training was the only factor associated with better results one month after the intervention. At the individual level (intrasubject change), increasing age improved the results of the intervention, while being a woman was associated with worse results.

Currently, medical students and young physicians have grown up with a level of technological literacy that was unthinkable just two decades ago. Educational providers in health care should take this into account when promoting undergraduate and graduate education, as well as continuing professional development. Games could be another tool for learning and practice.

## Supplementary Information

**Additional file 1.**

**Additional file 2.**

**Additional file 3.**

**Additional file 4.**

**Additional file 5.**

## Data Availability

The datasets used and/or analysed during the current study are available from the corresponding author upon reasonable request.

## Abbreviations

CI: Confidence interval
CPG: Clinical practice guideline
DE: Design effect
SD: Standard deviation
EBM: Evidence-based medicine
EPOC: Effective practice and organization of care
ICT: Information and communication technologies
TUs: Teaching units
UDMAFYC: Family and community care multidisciplinary teaching unit
