# Supplementary material for: Effectiveness of a game-based educational strategy e-EDUCAGUIA for implementing antimicrobial clinical practice guidelines in family medicine residents in Spain: a randomized clinical trial by cluster
Source: BMC Med Educ. 2022 Dec 24;22:893. doi: 10.1186/s12909-022-03843-4 (PMC9789537; doi:10.1186/s12909-022-03843-4)
Supplement: Supplementary file 3 — Additional file 3. [file 12909_2022_3843_MOESM3_ESM.docx]

**
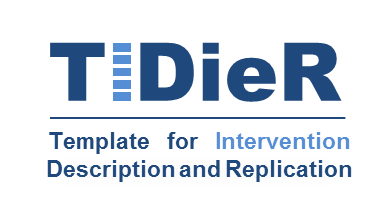
**

**The TIDieR (Template for Intervention Description and Replication) Checklist:**

| **BRIEF NAME** | EDUCAGUIA: effectiveness of an educational game for improving the knowledge of residents in MFyC (family and community medicine) regarding decision-making based on the recommendations of a clinical practices guidelines (CPG) on antimicrobial therapeutics. |
| --- | --- |
| **WHY** | Despite the potential of CPGs as teaching tools in the training of health care professionals, their implementation is still deficient. For a CPG to improve health care outcomes among medical specialists in training, an implementation strategy is necessary.  In recent years, educational games have been incorporated as a new strategy with potential in this field and are proposed as a new teaching methodology in physician training. However, current evidence on the effectiveness of educational games versus other conventional methodologies to improve knowledge and skills is limited. |
| **WHAT (materials)** | The e-Educaguia educational strategy, a quiz-like two-stage educational game in which experience is followed by reflection. |
| **WHAT (procedures)** | An initial 60-minute session was held for both groups (control and intervention) on what CPGs are and where to find them, the presentation of the GuiaSalud website and the CPG library. The initial session included an initial assessment of baseline knowledge of the guidelines. This test was repeated one month after the intervention. The procedures for each group were as follows:   - Control group: routine dissemination by e-mail of the CPG for infectious diseases in Aljarafe, included in the GuiaSalud catalogue. - Intervention group: routine dissemination of the guide by e-mail with an invitation to use the e-Educaguia educational strategy (the educational game designed for this project). Access to the application was provided for 1 month. |
| **WHO** | The e-Educaguía educational game was designed by a multidisciplinary team of primary care professionals with experience in training students and residents; professionals with experience in the development of CPGs; and professionals with experience in the development of new technologies as teaching tools.  The study included 200 MFyC residents from the 7 teaching units of the Community of Madrid who had been in training for at least 6 months. |
| **HOW** | Two face-to-face sessions were held for both groups: the initial one and another one a month after the intervention. The CPG was disseminated via e-mail and the GuiaSalud website, and the link to the CPG library was presented to allow each participant to access these resources.  In addition, the intervention group was given access to the e-Educaguía application during the month of the intervention. |
| **WHERE** | The initial and final sessions were held in the respective teaching units. |
| **WHEN AND HOW MUCH** | Two sessions were provided for all participants: one prior to the start of the study and another after one month. Both sessions included an assessment of knowledge of the CPG. During the month of the study, the intervention group had access to the e-Educaguía application. |
| **TAILORING** | Prior to the finalization of the e-Educaguía application, a pilot study was tested with 10 professionals (physicians and residents) on 4 different occasions. Their contributions were used to modify the questions and rules for the final version.  During the first week of use of the E-Educaguia application, the participants were consulted on possible improvements, which allowed us to correct the fact that the stopwatch did not stop when the participant requested a tutor's help. The fact that the time continued to count down constituted a disadvantage. |
| **MODIFICATIONS** | No modifications to the planned intervention occurred during the course of the study. |
| **HOW WELL (planned)** | A total of 46.2% of the participants in the intervention group used the game during the study month. Adherence was related to a higher number of hours of EBM training.  The posttest was completed by 100 residents: 45 in the intervention group (43.3%) and 55 in the control group (56.1%). |
| **HOW WELL (current)** | The participants in the intervention group provided input on the usability of the application both at the final session and by e-mail. This allowed some modification to be made during the study period. |
