# Supplementary material for: Effectiveness of a game-based educational strategy e-EDUCAGUIA for implementing antimicrobial clinical practice guidelines in family medicine residents in Spain: a randomized clinical trial by cluster
Source: BMC Med Educ. 2022 Dec 24;22:893. doi: 10.1186/s12909-022-03843-4 (PMC9789537; doi:10.1186/s12909-022-03843-4)
Supplement: Supplementary file 4 — Additional file 4. [file 12909_2022_3843_MOESM4_ESM.pdf]

e-educaguía

# Juegos de práctica clínica

Ayuda Salir

## Experto Recomendaciones de Guías de Práctica Clínica

### Seleccione área de conocimiento

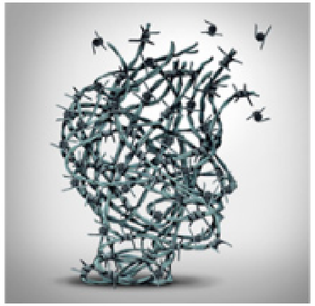

Ansiedad

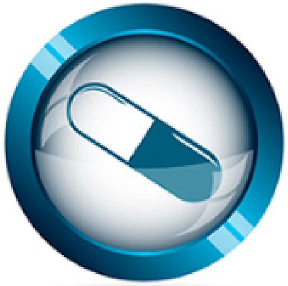

Antibioterapia

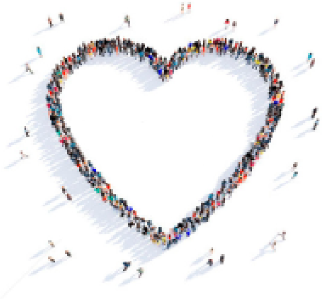

Cardiovascular

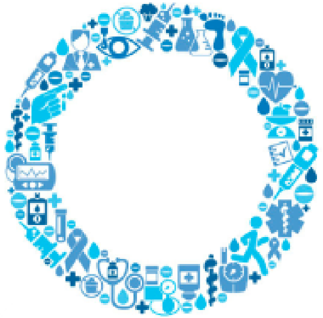

Diabetes

## Ranking Nacional

| | |
|---|---|
| ★★★★★ 03042 | 840 puntos |
| ★★★★ 01034 | 550 puntos |
| ★★★ 03072 | 550 puntos |
| ★★ 03003 | 510 puntos |
| ★ 01001 | 460 puntos |

## Competencias alcanzadas

**Warning**: count(): Parameter must be an array or an object that implements Countable in **/homepages/41/d599605878/htdocs/educaguia/wp-content/plugins/wmdgame/wmdgame.php** on line **135**

experto

exacerbación EPOC

experto

infección cutánea

experto

faringoamigdalitis

e-educaguía

# Juegos de práctica clínica

Volver Ayuda Salir

## Antibioterapia

### Experto en Antibioterapia

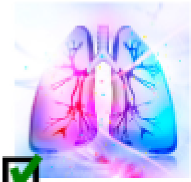

Exacerbación EPOC

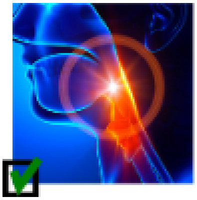

Faringoamigdalitis

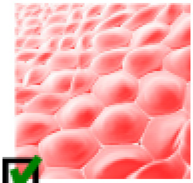

Infecciones cutáneas

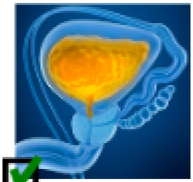

Infecciones del Tracto Urinario

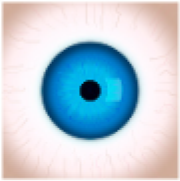

Infecciones Oftalmológicas

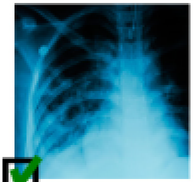

Neumonía

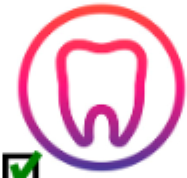

Odontalgia

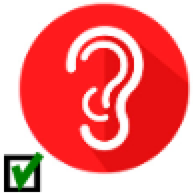

Otitis

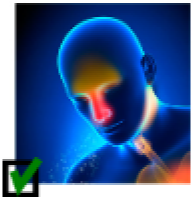

Sinusitis

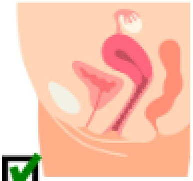

Vaginitis

### Ranking Nacional

| | |
|---|---|
| ★★★★★ 03042 | 840 puntos |
| ★★★★ 01034 | 550 puntos |
| ★★★ 03072 | 550 puntos |
| ★★ 03003 | 510 puntos |
| ★ 01001 | 460 puntos |

### Competencias alcanzadas

Warning: count(): Parameter must be an array or an object that implements Countable in /homepages/41/d599605878/htdocs/educaguia/wp-content/plugins/wmdgame/wmdgame.php on line 135

experto exacerbación EPOC

experto infección cutánea

experto faringoamigdalitis

experto infecciones odontológicas

experto neumonía

experto infecciones del tracto urinario

experto sinusitis

e-educaguía

# Juegos de práctica clínica

Volver Ayuda Salir

Ranking Nacional

★★★★★ 03042 840 puntos
★★★★ 01034 550 puntos
★★★ 03072 550 puntos
★★ 03003 510 puntos
★ 01001 460 puntos

Tiempo

355"

Puntos: 0

Comodines

- Pregunta al tutor
- 50%
- Quiero leerme la guía

Paciente de 20 años que acude porque presenta picor, dolor y pequeñas lesiones en región inguinal derecha tras depilarse hace 5 días. No refiere fiebre ni ninguna otra sintomatología. No presenta ningún antecedente personal ni familiar de interés. A la exploración presenta pequeñas pápulo-pústulas pruriginosas con una "cabeza de pus" centrada en varios folículos pilosos. ¿Cuál es el diagnóstico más probable que presenta esta paciente?

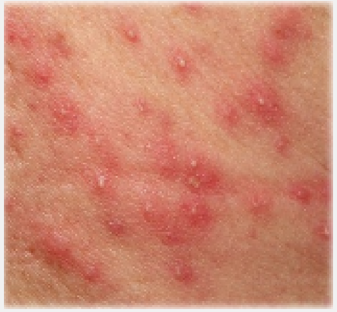

- ○ Forúnculo
- ○ Foliculitis
- ○ Ántrax
- ○ Celulitis

Enviar

Competencias alcanzadas

**Warning**: count(): Parameter must be an array or an object that implements Countable in **/homepages/41/d599605878/htdocs/educaguia/wp-content/plugins/wmdgame/wmdgame.php** on line **135**

experto — exacerbación EPOC

experto — infección cutánea

experto — faringoamigdalitis

experto — infecciones odontológicas

experto — neumonía

e-educaguía

# Juegos de práctica clínica

Volver Ayuda Salir

Ranking Nacional

★★★★★ 03042 — 840 puntos
★★★★ 01034 — 550 puntos
★★★ 03072 — 550 puntos
★★ 03003 — 510 puntos
★ 01001 — 460 puntos

Tiempo

Puntos: 0

Comodines

- Pregunta al tutor
- 50%
- Quiero leerme la guía

Este ejercicio se realizó, con las siguientes respuestas:

Paciente de 64 años con los siguientes antecedentes *** que acude a la consulta porque en los últimos 5 días presenta aumento de su disnea habitual hasta hacerse de mínimos esfuerzos, con ortopnea asociada, aumento de su tos habitual, cambio del aspecto de la expectoración, siendo más abundante y de color verdosa más fiebre de 38,3º en las ultimas 24 horas. ¿Cuáll es el grado de obstrucción de este paciente EPOC? *** Abrir con hipervínculo: - Exfumador (fumador hasta hace 5 años con IPA= 55) - EPOC diagnosticado hace 9 años.

• Tratamiento continuado con Bromuro de Ipratropio(2 inh/ 8 horas) y formoterol12 mcg (inh/12 horas) +/- Corticoides inhalados de forma ocasional en algunas reagudizaciones. • Espirometría basal (10 meses antes): FCV:3420 (65%); FEV1:1820 (63%); FEV1/FCV: 51%. • Ha sufrido 3 exacerbaciones en el último año (última hace 5 meses), sin necesidad de hospitalización. • Situación basal: Tos y expectoración crónica habitual. Disnea grado II (de moderados esfuerzos) . - HTA en en tratamiento con enalapril e HCT (20/12,5 mg) - Hipercolesterolemia en tratamiento con simvastatina 20 mg/día. - Obesidad (IMC: 34). - Ingesta diaria de alcohol: 2 unidades al día.

- Leve
- Moderado
- Grave
- Muy grave

El paciente desde hace 2 días se ha automedicado con amoxicilina (500 mg/8 h), pero no ha mejorado En la exploración física destaca: - Ctes: 38,1°C, 92% sat , 24 rpm, 76 lpm, 140/70. - AC: normal. - AP: ** que aparezca una espalda con un fonendo y que al pulsar sonara: Roncus y sibilantes dispersos por ambos campos + Diminución generalizada del MVC El cuadro clínico del paciente parece compatible con una exacerbación de causa infecciosa, ¿de los siguientes cuáll sería el agente etiológico más probable que presenta este paciente?

- Pseudomona Aeruginosa
- Polimicrobiana
- Rinovirus
- Haemophilus influenzae

## Competencias alcanzadas

**Warning**: count(): Parameter must be an array or an object that implements Countable in **/homepages/41/d599605878/htdocs/educaguia/wp-content/plugins/wmdgame/wmdgame.php** on line **135**

experto — exacerbación EPOC

experto — infección cutánea

experto — faringoamigdalitis

experto — infecciones odontológicas

experto — neumonía
