# Supplementary material for: Effectiveness of a game-based educational strategy e-EDUCAGUIA for implementing antimicrobial clinical practice guidelines in family medicine residents in Spain: a randomized clinical trial by cluster
Source: BMC Med Educ. 2022 Dec 24;22:893. doi: 10.1186/s12909-022-03843-4 (PMC9789537; doi:10.1186/s12909-022-03843-4)
Supplement: Supplementary file 5 — Additional file 5. [file 12909_2022_3843_MOESM5_ESM.docx]

|  | **n** | **%** | **95% CI** |
| --- | --- | --- | --- |
| **UDMAFyC North** | 56 | 28.0% | 21.7–34.3% |
| **UDMAFyC West** | 61 | 29.5% | 23.1–35.9% |
| **UDMAFyC South** | 31 | 15.5% | 10.4–20.6% |
| **UDMAFyC Southeast** | 10 | 5.0% | 2.0–8,0% |
| **UDMAFyC Center** | 13 | 6.5% | 3.0–9,9% |
| **UDMAFyC Northwest** | 17 | 8.5% | 4.6–12.4% |
| **UDMAFyC East** | 14 | 7.0% | 3.4–10.6% |
|  |  |  |  |

**Supplement 5. Number of participants per teaching unit. UDMAFYC: Multidisciplinary Teaching Unit of Family and Community Care.**
